# Investigating Polypyrrole/Silver-Based Composite for Biofilm Prevention on Silicone Surfaces for Urinary Catheter Applications

**DOI:** 10.1021/acsomega.4c10109

**Published:** 2025-02-17

**Authors:** Maíra C. Marcolino, Milena L. Guimarães, Marina de L. Fontes, Flávia A. Resende, Hernane da S. Barud, Andreia S. Azevedo, Nuno F. Azevedo, Helinando P. de Oliveira

**Affiliations:** † LEIMO—Impedance Spectroscopy and Organic Materials Laboratory, Federal University of Vale do São Francisco (UNIVASF), Juazeiro 48902-300, Bahia, Brazil; ‡ RENORBIO—Northeast Biotechnology Network, Federal Rural University of Pernambuco (UFRPE), Recife 52171-900, Pernambuco, Brazil; § Federal University of São Carlos (UFSCar), São Carlos 13565-905, São Paulo, Brazil; ∥ University of Araraquara (Uniara), Araraquara 14801-340, São Paulo, Brazil; ⊥ LEPABE—Laboratory for Process Engineering, Environment, Biotechnology and Energy, Faculty of Engineering, University of Porto, Porto 4099-002, Portugal; # ALiCE—Associate Laboratory in Chemical Engineering, Faculty of Engineering, University of Porto, Porto 4099-002, Portugal

## Abstract

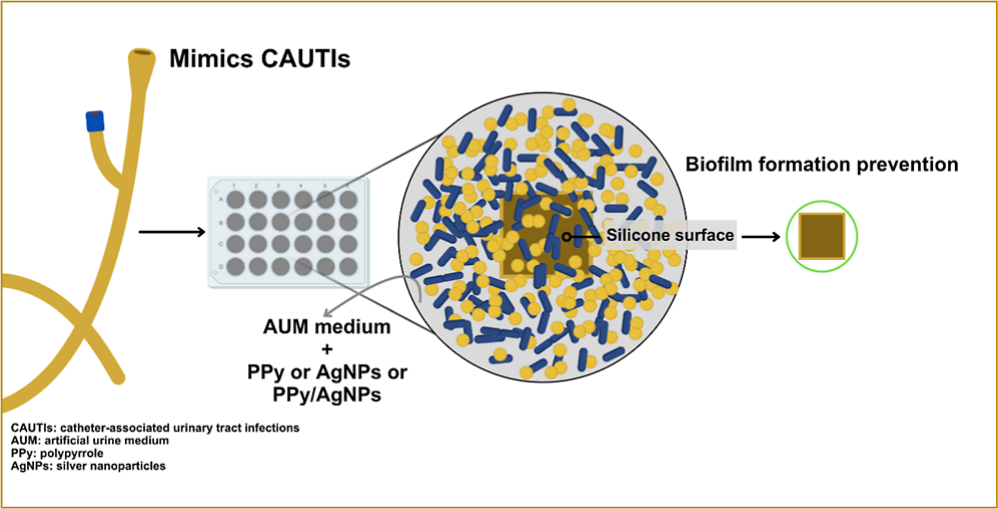

Catheter-associated urinary tract infections (CAUTIs) are among the most common healthcare-related infections caused by biofilm formation. This research investigated the efficacy of polypyrrole (PPy), silver nanoparticles (AgNPs), and their combination (PPy/AgNPs) as water-soluble additives applied in cleaning procedures for preventing the formation of *Escherichia coli* and *Staphylococcus aureus* (single and dual-species biofilms) on silicone. Ultraviolet–visible absorption assays, scanning electron microscopy (SEM) images, FTIR analysis, and dynamic light scattering experiments were conducted to evaluate the structure and physicochemical response of the antibiofilm compounds, with the biofilm prevention concentrations assessed by plate counting, flow cytometry, and SEM images. The composites proved to be dose-dependent agents preventing single- and dual-species biofilms from forming under simulated CAUTI conditions. Furthermore, cytotoxicity assays indicated that the materials are non-cytotoxic, supporting their suitability for biomedical applications. These findings pave the way for developing more effective, biocompatible catheter cleaning procedures, ultimately improving patient outcomes and addressing biofilms-related infections in clinical settings.

## Introduction

Using catheters in hospital settings is a resource for medical treatment in different systems of the human body.^1−3^ However, the available surface for microbial growth is frequently reported as a source of bacterial infection.^4−7^ CAUTIs are examples of nosocomial-related infections,^8,9^ caused by inadequate catheter disinfection since the presence of proteins and other nutrients in urine facilitates biofilm formation on the surface of urinary catheters, creating an environment conducive to bacterial adhesion.^8,10^ Given the intrinsic characteristics of biofilm formation on the catheter, bacterial strain multiplication occurs with microcolonies and the secretion of an extracellular matrix to encapsulate the cells.^11−13^ Within this matrix, the colonies continue to grow and release sessile cells and biofilm aggregates to sustain the infection cycle.^10^ It has been demonstrated that CAUTIs are polymicrobial, and their interactions result in virulence and microbial diversity in a biofilm.^14,15^*Escherichia coli* is reported as the primary pathogen in CAUTIs, followed by *Klebsiella* spp., *Pseudomonas aeruginosa*, *Candida* spp., and *Enterococcus* spp.^16−18^ On the other hand, although *Staphylococcus aureus* has a lower incidence of CAUTIs, it remains clinically relevant due to its ability to form biofilms favoring antibiotic resistance.^19^

The impact of a multispecies consortium in CAUTI-associated biofilms has been reported by Azevedo and collaborators,^20^ who studied the interactions between *E. coli* (typical), *Delfia tsuruhatensis*, and *Achromobacter xylosoxidans* (atypical). The authors observed that *E. coli* could form biofilms, even in the presence of preformed biofilms by the atypical species. Galván et al.^21^ investigated the interactions between *E. coli*, *K. pneumoniae*, and *Enterococcus faecalis* in dual-species biofilms, highlighting changes in initial adhesion, cell dispersion, and population composition. Furthermore, given the intricacy of the formations and interactions within polymicrobial biofilms, Allkja and collaborators^22^ demonstrated the complexity of interactions in biofilms formed by four species related to CAUTIs. The authors identified antagonistic interactions between *E. coli* and *Candida albicans*, mutualistic interactions involving *E. faecalis*, and the dominance of *Proteus mirabilis* over *E. coli*.

Consequently, more than conventional prevention and treatment methods may be required to prevent and control these infections. The initial bacterial adhesion process might be disrupted by developing novel approaches, such as surface treatment of the catheters, thereby reducing biofilm development and subsequent infection. For instance, Dave et al.^23^ studied the influence of biofilm formation on physicochemically altered silicone catheter surfaces. The authors confirmed the efficiency of the oxygen plasma-treated surface in reducing bacterial adhesion by 99.4%. Elzahaby et al.^24^ functionalized the surfaces of urinary silicone catheters using gamma irradiation and incorporated zinc nanoparticles. As a result, the authors confirmed the biocompatibility and self-anti-biofouling activity of the modified catheter. Wynne et al.^25^ achieved relevant results regarding antimicrobial activity through direct contact with microorganisms by applying *N*,*N*-dimethyl tetradecyl amine to modify biomedical silicone tubes. Polypyrrole (PPy), a conducting polymer, offers promising properties that enhance the antimicrobial properties of catheters.^26,27^ The cationic surface of polypyrrole favors the electrostatic interaction with oppositely charged species (such as the bacterial cell wall), making the PPy a promising antibacterial agent, as previously reported by our group.^28^ These advancements could significantly improve the prevention of biofilm-associated infections, especially in the face of polymicrobial communities.

Due to its biocompatibility, PPy has been used to develop micromachined catheters for enhanced intravascular imaging and navigation.^29,30^ PPy has been considered a valuable material for preventing oral biofilm-associated diseases due to its physical and electrochemical properties by inactivating *Streptococcus mutans* GTFs.^31^ PPy has also been analyzed in its pristine form and combined with other polymeric or metallic materials, such as silver nanoparticles (AgNPs), for evaluating antibacterial, antibiofilm, and healing activities.^26,32−34^ Divya et al.^35^ reported that biosynthesized AgNPs from coral-associated bacteria demonstrated strong antibiofilm and antimicrobial activity when applied in catheters coated with these nanoparticles. AgNPs have also been employed with other materials to modify silicone urinary catheters. Liu et al.^36^ combined amphiphilic carbonaceous particles with AgNPs to control biofilms formed by gram-positive and gram-negative bacterial strains. By using silver and zinc to functionalize catheter surfaces, Vaitkus et al.^37^ confirmed the prevention of biofilm formation for at least 6 days.

However, PPy and AgNPs’ combined activity against polymicrobial biofilms remains unexplored. Given the promising properties of PPy, such as its stability and biocompatibility, combined with the well-documented antibiofilm activity of AgNPs, this synergistic approach can be considered for improving biofilm prevention. Herein, the effect of soluble freeze-dried PPy (sodium dodecyl sulfate-coated PPy), AgNPs, and their composite was evaluated in the prevention of single- and mixed-species biofilms formation on silicone surfaces is investigated using artificial urine to mimic CAUTI conditions.

Based on the high solubility in water of the resulting components (PPy and PPy/AgNPs), the novelty of this paper is the proposal of a simple strategy for inhibiting biofilm formation on the silicone surface by a cleaning procedure in which the dispersion of compounds in water is sufficient to avoid the formation of biofilm on the catheter surface. To this end, viable cells were evaluated through plate counting, total cells through flow cytometry, and the cytotoxicity of the composites to ensure their safety in medical applications.

## Results and Discussion

### Physicochemical Characterization of Soluble PPy, AgNPs, and Their Interaction

**Figure 1:**
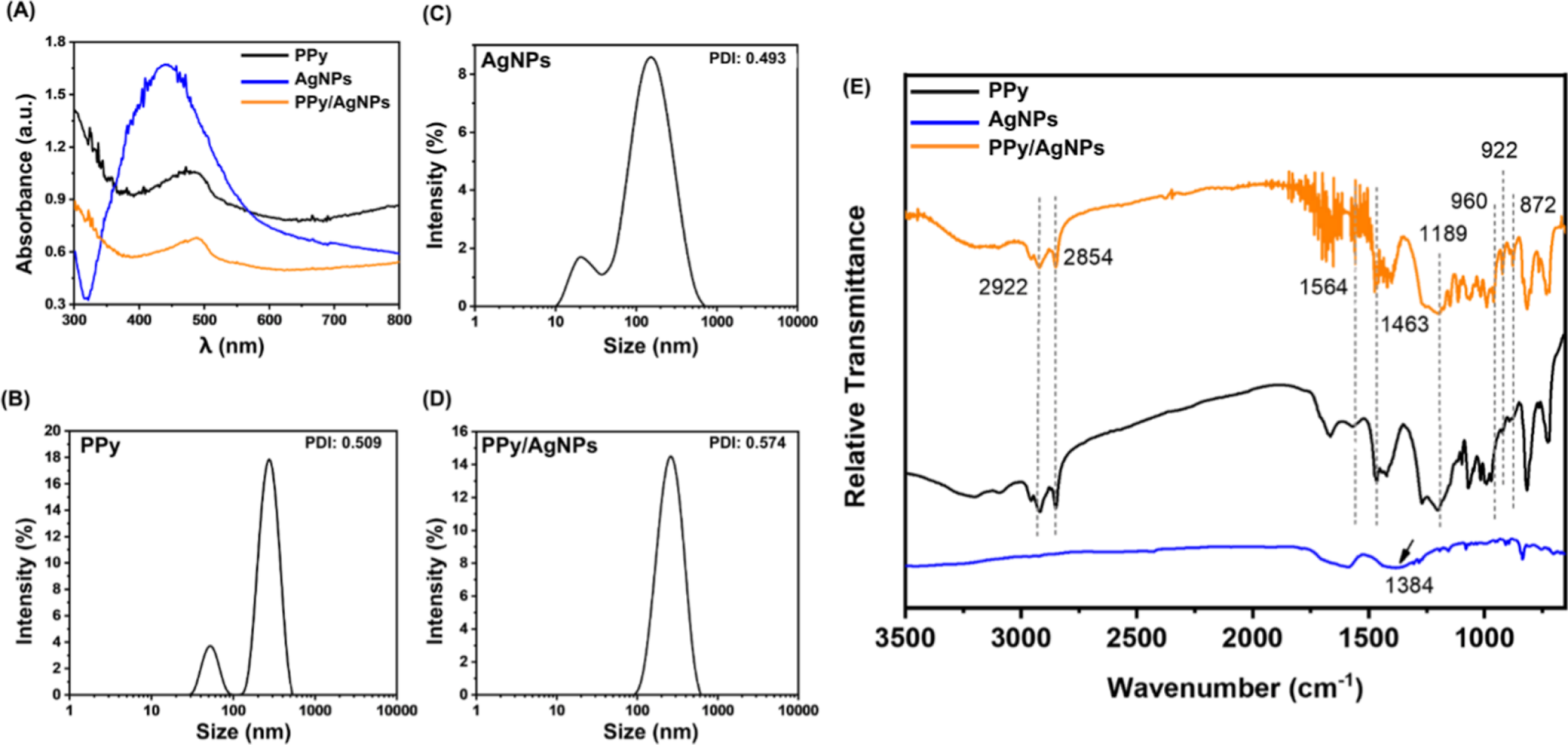
Optical and vibrational characterization of materials: (A) ultraviolet–visible (UV–vis) spectrum of absorbance of materials; particle size distribution by the dynamic light scattering technique of materials: PPy (B), AgNPs (C), and PPy/AgNPs (D) and FTIR spectrum of PPy, AgNPs, and PPy/AgNPs powders (E).

The physicochemical properties of the antimicrobial agents were evaluated as follows: the UV–vis absorption spectra of the chemically synthesized PPy, AgNPs, and PPy/AgNPs (core–shell structures of coated AgNPs by PPy) are shown in Figure 1A. The absorption band at 486 nm for PPy indicates a π–π* transition between molecular and antibonding orbitals, with a positive slope toward longer wavelengths, characteristic of its doped form,^26,38,39^ indicating the excitation of electrons and the conjugated bonds in the polymer structure. For AgNPs, a single band with maximum absorbance was observed at 441 nm, characteristic of the surface plasmon resonance (SPR) band at this wavelength.^39^ The PPy/AgNPs composite response confirms the doped state with a positive slope and a broad band around 800 nm. There is also a slight red shift in the characteristic PPy peak (shifted to 490 nm), suggesting the aggregation of AgNPs domains with PPy nanostructures. The structure of AgNPs, through changes in size and shape, affects this optical behavior, as reported in the literature.^38,40^ Regarding the morphology of AgNPs and their influence on the absorbance spectrum, and in agreement with the findings reported by Pal et al.,^41^ a single band in the absorbance spectrum for spherical nanoparticles is observed for AgNPs reduced by sodium citrate at 410–450 nm range. The suppression of the plasmonic band for the composite can be attributed to the low absorption intensity of AgNPs, which is overshadowed by the absorption band of polypyrrole (the coating layer on AgNPs).^26,38,39^

Regarding the size distribution of the nanoparticles, dynamic light scattering (DLS) suggested the existence of two populations of PPy particles with an average diameter of 176 nm (Figure 1B). The AgNPs exhibited a polydisperse distribution with an average size of 187 nm (Figure 1C). The PPy/AgNPs composite (280 nm) returned a homogeneous distribution of particles (Figure 1D). The redshift in the UV–vis spectrum justifies the nanocomposites’ increased nanoparticle size, indicating the composite’s aggregation stages.^40,42^

The structure of the composites was examined using FTIR spectra in the range of 3500 to 650 cm^–1^ (Figure 1E). The characteristic groups of PPy were observed with a band at 2922 cm^–1^, indicative of the C–H stretching vibration.^43^ The band at 2854 cm^–1^ is attributed to the symmetric overlapping vibration of C–H (in CH_3_ and CH_2_ functional groups); in the spectra of PPy and PPy/AgNPs samples, the peak at 1564 cm^–1^ is assigned to the conjugated C=C stretching vibration.^32^ The band around 1463 cm^–1^ is attributed to the vibrations of the aromatic rings typical of PPy.^44^ The band at 1189 cm^–1^ can be attributed to the pyrrole ring breathing vibration, while the peaks around 960, 922, and 872 cm^–1^ are attributed to the C–H vibration, characteristic of the pyrrole ring stretching.^43,45^ On the other hand, the band observed at 1384 cm^–1^ in the AgNPs spectrum refers to the N–O stretching vibration, indicating doping with the nitrate group since AgNO_3_ was the silver source for the nanoparticle synthesis.^27,46^

**Figure 2:**
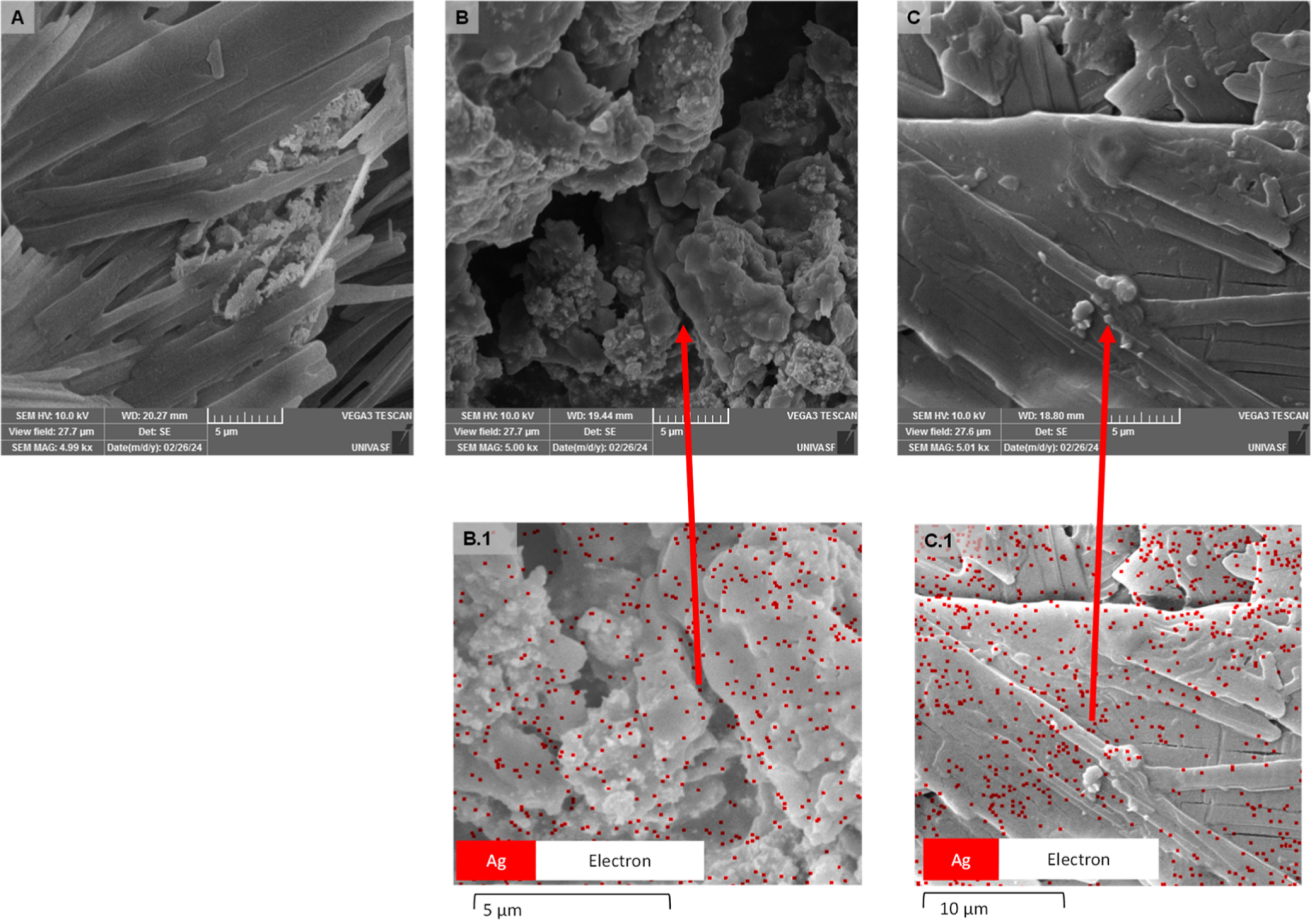
SEM images (magnification of 5k×) for freeze-dried PPy (A), composites of PPy/AgNPs (B and C), and overlaid EDS images for identification of Ag elements as red dots (B.1 and C.1).

The morphology of synthesized polypyrrole (after the freeze-drying process) prevails as dispersion fibers and grains of the conducting polymer (as shown in Figure 2A). The alignment of polymer chains between ice clusters favors the polypyrrole fibers formation under water sublimation. Figure 2B,C show the granular aspect in the border and on the surface of PPy/AgNPs composites. The identification of silver element is provided by energy dispersive X-ray spectrometer (EDS) overlaid mappings (shown in Figure 2B.1,C.1) in which red dots characterized the local distribution of identified silver element—homogeneously dispersed into the polymeric matrix.

### Effect of PPy, AgNPs, and PPy/AgNPs on Biofilm Formation Prevention and Cytotoxicity

The antibiofilm efficacy of the composites was evaluated by determining the biofilm prevention concentration (BPC).^47,48^ BPC values for *E. coli* were 750, 90, and 500 μg/mL for PPy, AgNPs, and PPy/AgNPs, respectively, while for *S. aureus*, these values were 500, 60, and 500 μg/mL, respectively. Therefore, subsequent analyses were performed using concentrations of 1/2× BPC and 1× BPC obtained against *E. coli* for both bacterial species. The effects of the composites and their respective concentrations were analyzed on biofilms using the plate count method (for culturable cell counts, CFU/cm^2^), flow cytometry (for total cell counts/cm^2^), and scanning electron microscopy (SEM). Additionally, for *S. aureus*/*E. coli* dual-species biofilms, the CFU/cm^2^ values were converted into percentages from the quantification of the relative abundance of the populations, allowing the identification of the prevalence of each bacterial strain on the composites.

**Figure 3:**
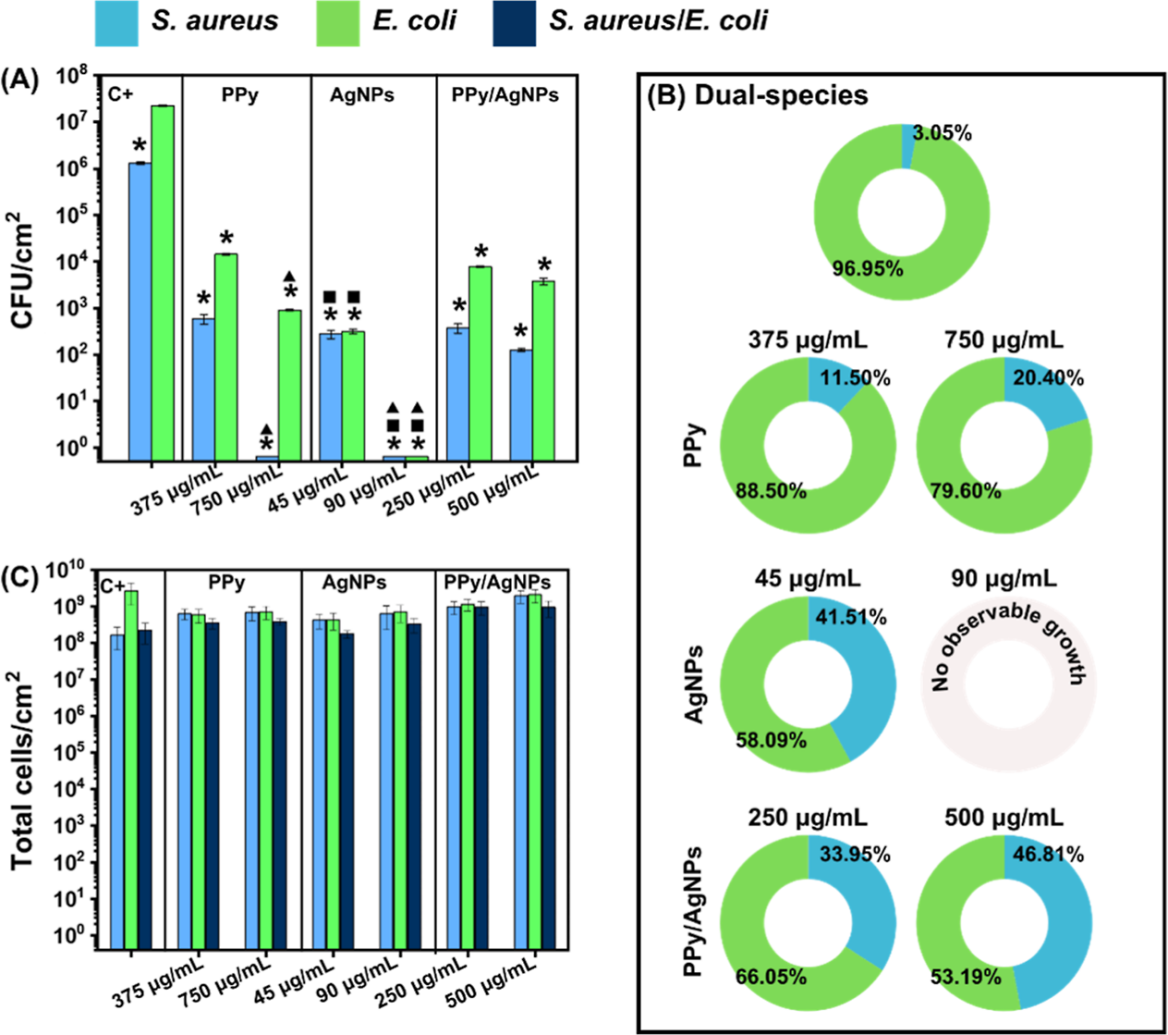
Antibiofilm effect of PPy, AgNPs, and PPy/AgNPs: (A) quantification of culturable cells from single-species biofilms (*S. aureus* and *E. coli*); (B) relative distribution of bacteria in dual-species biofilms; (C) total cells from single- and dual-species biofilms. Values represent the mean ± standard deviation. Significant differences (*p* < 0.05) between positive controls, composites, and concentrations were represented by *, ■, and ▲, respectively.

Initially, single and dual-species biofilms were analyzed to confirm the ability of *S. aureus* and *E. coli* to form biofilms on silicone surfaces under conditions that mimic CAUTIs. In this context, single-species biofilms showed statistically significant differences (*p* < 0.05), with *E. coli* exhibiting a higher number of culturable cells (log 7.3 CFU/cm^2^) compared to *S. aureus* (log 6.1 CFU/cm^2^) (Figure 3A). Similarly, in the dual-species consortium, the population of *E. coli* (log 6.1 CFU/cm^2^) was significantly higher than that of *S. aureus* (log 4.6 CFU/cm^2^) (Figure 3B). Despite *E. coli* maintaining its population dominance, these data suggest that under consortium, the presence of *E. coli* negatively affected *S. aureus* biofilm formation, possibly due to competition for resources or the production of substances that inhibit *S. aureus* growth. Furthermore, the prevailing *E. coli* population makes it more competitive or resilient than *S. aureus* in the biofilm environment. Regarding total cells (Figure 3C), no significant differences (*p* < 0.05) were observed between single and dual-species biofilms; the total cells/cm^2^ was log 9.4 for *E. coli*, log 8.2 for *S. aureus*, and log 8.3 for dual-species biofilms, with this value being consistent with the counts of culturable cells, which also showed a reduction in their populations under consortium.

Maharjan et al.^49^ observed similar results for monospecies biofilms, in which *E. coli* was identified as the most frequent uropathogenic (57%) and the highest biofilm producer. *S. aureus* reached a value of 8%, characterized as a weak biofilm producer. For *E. coli*, these results are consistent with previous findings, even in pre-existing microbiota or interaction with less common species in CAUTIs.^20,50,51^ On the other hand, the recurrence of *S. aureus* as a weaker biofilm-forming strain, both individually and in combination with gram-negative bacterial species, has also been reported in other studies that mimic CAUTI conditions.^51,52^ The competitive behavior in dual-species biofilms between *S. aureus* and *E. coli* has been previously identified, with *E. coli* being characterized as the more competitive species with a greater proliferative capacity.^53,54^ In this regard, Rendueles et al.^55^ confirmed the presence of anti-adhesion molecules in *E. coli* biofilms, which possess antibiofilm activity against different gram-negative and gram-positive strains, including *S. aureus*. Additionally, the authors isolated the anti-adhesion polysaccharide Ec300 from *E. coli*, which was characterized by hindering the initial interactions in mixed biofilms of *S. aureus* and *E. coli*. Wong and collaborators^56^ identified that coinfection of *E. coli* with *S. aureus* induces the synthesis of colibactin by the pks island genes, which is detrimental to *S. aureus* and is regulated by the BarA-UvrY two-component system (TCS) under interspecies competition. The authors also observed that nutrient competition in the bacterial consortium could reduce the number of *S. aureus* colonies by one or two logs compared to the cell count of the monospecies, which agrees with the behavior of dual-species biofilms.

Regarding the response of antibacterial components (Figure 3A), the results suggest that the effect on biofilm formation prevention varies significantly for *S. aureus* and *E. coli* in comparison with the positive control (*p* < 0.05)—due to the influence of polypyrrole—as observed for the isolated contribution of the component. The cultivability values of the single-species biofilms of *E. coli* remained higher than those of *S. aureus*, except for the AgNPs, in which equilibrium in the biofilm formation prevention efficacy was observed for both bacterial strains. Among the antibiofilm agents, AgNPs were also the most effective in reducing the culturable cells of *S. aureus* and *E. coli* (*p* < 0.05), as were the concentrations of 750 μg/mL of PPy and 90 μg/mL of AgNPs. A similar trend was noted for the dual-species biofilms (Figure 3B), in which *E. coli* could dominate the co-culture. However, it was the most sensitive species to the composites, while the population of *S. aureus* increased even at the highest dose of PPy. These results suggest a possible adaptive expression of the populations to the new stress conditions.^50^ The assessment of the total cells in the single- and dual-species biofilms did not show significant differences (*p* < 0.05) in cell reduction for any of the composites (Figure 3C).

An important aspect to consider for PPy-based samples is the intense activity against *S. aureus* (isolated and combined), which is confirmed by the complete elimination of *S. aureus* at 750 μg/mL and the reduction in the relative content of *S. aureus* in dual-species contaminations. The adequate control of the relative concentration of AgNPs in the PPy matrix can be further explored to achieve outstanding composite performance with minimal content of both materials.

**Figure 4:**
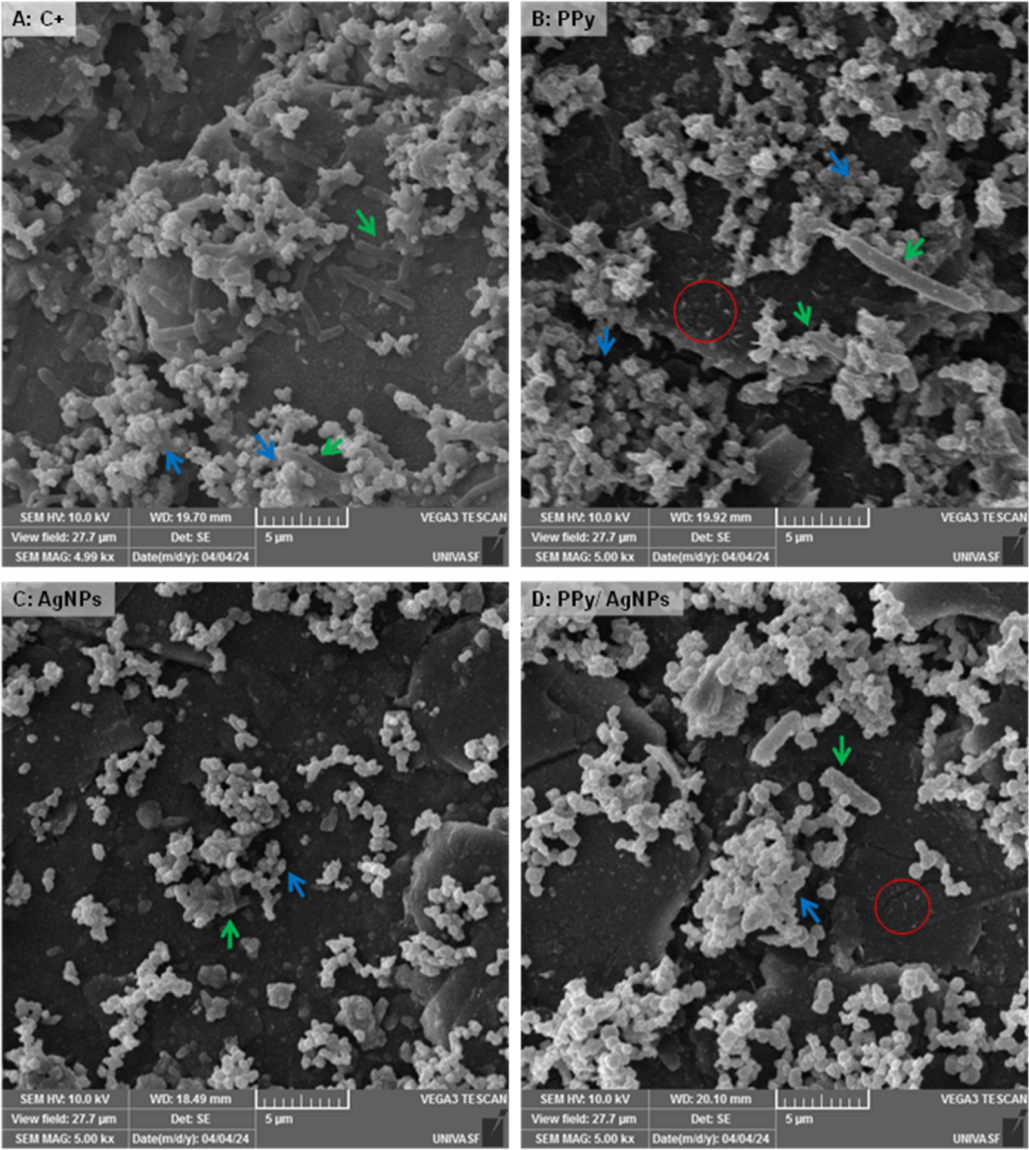
SEM analysis of dual-species biofilms of *S. aureus* (blue arrow) and *E. coli* (green arrow) formed on silicone coupons after 24 h of incubation. (A): positive control; (B): treated with PPy at 750 μg/mL; (C): treated with AgNPs at 90 μg/mL; (D): treated with PPy/AgNPs at 500 μg/mL. Red circles indicate extracellular matrix or extravasated intracellular material. Magnification: 5 k×.

The cell morphology and biofilm organization under the action of the composites at the highest concentrations, as well as control biofilms, were evaluated by SEM analysis (Figure 4), which provides qualitative information about the structure and morphology of bacteria in the biofilm. For positive control biofilm on the silicone surface (Figure 4A), *E. coli* cells are deposited in a monolayer and incorporated into clusters of *S. aureus*. In contrast, *S. aureus* dominates the upper layer by forming aggregates. This conformation was observed by Barros et al.^53^ for the same bacterial species in a dual-species biofilm formed on a nanohydroxyapatite (nanoHA) surface. It was also possible to identify layers of extracellular polymeric substances (EPS) above the cell clusters, a phenomenon related to biofilm formation over 24 h, confirming the vulnerability of silicone catheter surfaces to bacterial biofilm formation.

Figure 4B,D showed the presence of deformed bacterial cells in biofilms treated with PPy and PPy/AgNPs. Furthermore, possible fragments related to the degradation of the extracellular matrix or extravasated intracellular material could be observed on the surface with these composites (red circle). Fragments for biofilm treated with AgNPs were not observed, which may be related to different mechanisms of antibiofilm action. In samples with AgNPs (see Figure 4C), a cell number reduction was observed, particularly in *E. coli* and *S. aureus* aggregates, corresponding to the results of the culturable cell counts, where colony formation was inhibited entirely, suggesting that prevailing dead cells are observed in the biofilm in agreement with the counting data (in cells/cm^2^).

To our knowledge, this is the first study about the influence of water-soluble PPy and their composite with AgNPs in dual-species biofilms, mimicking the conditions observed in CAUTIs. From this perspective, and in the context of biological applications, PPy nanoparticles can avoid biofilm deposition.^57^ Additionally, the use of PPy as an efflux pump inhibitor against drug-resistant *S. aureus* strains has been reported.^58^ Regarding antibiofilm activity, PPy has been studied alone or in combination with other compounds. Wang et al.^34^ developed a coating based on tannic acid, poly(vinyl alcohol), and PPy (TA/PVA-PPy) for photothermal treatment and observed reduced bacterial adhesion and effective prevention of biofilm formation by *E. coli* and *S. aureus*. On the other hand, AgNPs are versatile due to their high surface area, making them an attractive, stable, and clinically available option for incorporation into medical devices. The release of silver ions (Ag^+^) causes oxidative stress in bacterial cells and results in cell membrane damage, in addition to their ability to inhibit biofilm formation by *E. coli* and *S. aureus*.^35,36,59^

Studies have reported that silver-coated catheters effectively reduce the incidence of CAUTI; however, they also suggest that urinary catheters with silver could be improved by incorporating other substances.^37^ It is worth mentioning that strategies reported in the literature for the effective development of antibiofilm agents involve the surface modification of the silicone-based material. Herein, the reported results were acquired from the dispersion of composites into the aqueous solution (typical cleaning additives), avoiding the additional steps of the catheter surface modification.

**Figure 5:**
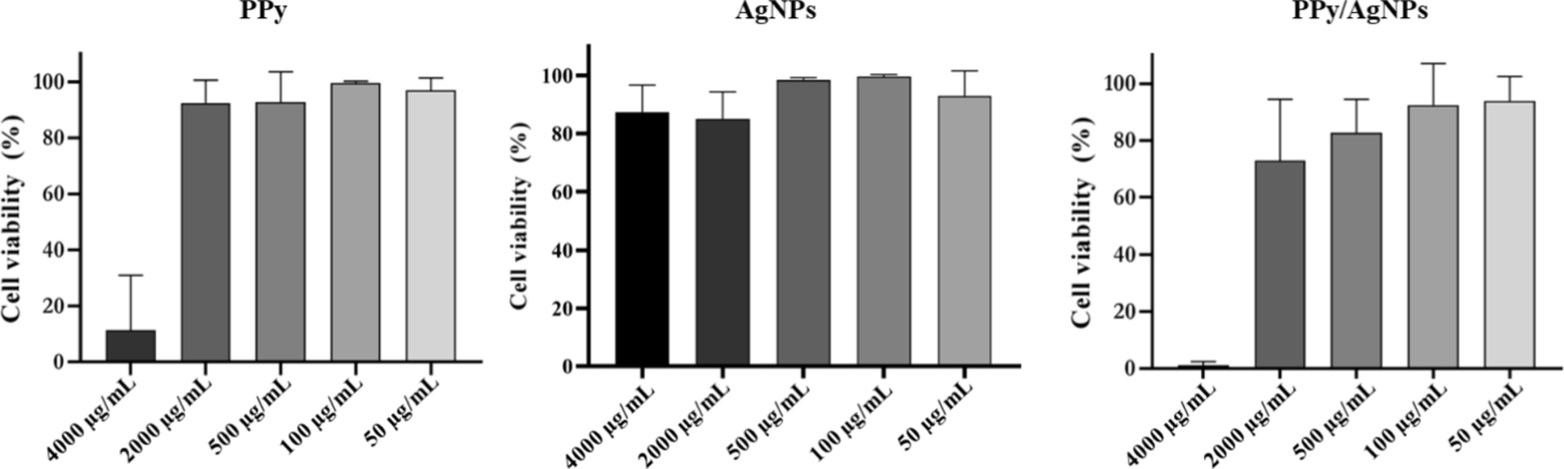
Evaluation of the cytotoxicity of different concentrations of PPy, AgNPs, and PPy/AgNPs (4000 to 50 μg/mL) in L929 cells. Cell viability (%) was determined using a colorimetric assay using MTT.

The composites’ cytotoxicity against murine fibroblasts was analyzed according to ISO 10993-5,^60^ which states that cell viability must be greater than 70% for materials/devices intended for medical applications. The maximum concentrations tested in this study for antibiofilm activity were 750, 90, and 500 μg/mL for PPy, AgNPs, and PPy/AgNPs, respectively, in which negligible cytotoxic (cell viability > 70%) was observed for all of them (Figure 5).

Polypyrrole derivatives are commonly reported to be biocompatible; however, this characteristic can vary depending on the polymer synthesis method and the cell line used for cytotoxicity evaluation.^61^ Under testing the cytotoxicity of PPy/PVP nanoparticles on the L929 cell line, Guo et al.^62^ defined 100 μg/mL as a noncytotoxic concentration. Conversely, Káčerová et al.^63^ using NIH/3T3 embryonic fibroblast cells and testing colloidal PPy, also stabilized with PVP, indicated 200 μg/mL as a safe concentration for biomedical applications. Hermenegildo et al.^64^ developed membranes for biomedical applications, and PPy was applied to electrospun fiber coatings. In their cytotoxicity assessment, these fibers showed a cell viability potential above 70% (L929), in agreement with the data presented in this study, even at concentrations up to 2000 μg/mL.

Similarly to studies involving polypyrrole, differences in the cytotoxicity of AgNPs depend on the synthesis method, chemical precursors, and corresponding effects on the particle size.^65^ Liu et al.^66^ evaluated polyetheretherketone coatings with silver nanoparticles and reported negligible cytotoxic activity. Lethongkam et al.^67^ reported biocompatibility in a urinary catheter coated with AgNPs, even after 72 h of exposure. Składanowski et al.,^68^ through biosynthesis of AgNPs, found preserved cell viability up to a 25 μg/mL concentration. In this study, there was no reduction in cell viability, even at a concentration of 4000 μg/mL. However, despite the benefits of AgNPs, continuous exposure has also been reported to result in bacterial resistance and dose-dependent cytotoxicity.^69^ Therefore, it is crucial to emphasize the importance of physical–chemical and biological evaluations for composites intended for biomedical applications. Furthermore, although this study does not include an environmental assessment, the release of AgNPs into natural environments is an emerging concern in materials science, primarily due to the toxicity resulting from the continuous release of Ag^+^ ions.^70^ To circumvent drawbacks related to bioaccumulation and ecotoxicity of high dosage of AgNPs in the control of biofilm development, the association with polypyrrole represents a promising aspect in which the relative concentration of AgNPs into the composite is critical, given the desirable performance, combining the role of the polymeric support (PPy) with outstanding effect against *S. aureus* (isolated or combined species). As a perspective for this study, the evaluation of the surface parameters in silicone can be considered from surface energy, hydrophilicity, and corresponding assays to provide a complete description of the surface protection offered by the nanostructures.

## Conclusions

This study provides a strategy for producing polypyrrole (PPy), silver nanoparticles (AgNPs), and their PPy/AgNPs composite applied as antibiofilm agents under simulated conditions of CAUTIs. The results revealed that both PPy and AgNPs exhibited significant efficacy in preventing biofilm formation, with AgNPs emerging as the most effective agent in both monospecies and dual-species biofilms. Conversely, polypyrrole inhibits the *S. aureus*-based biofilm formation more efficiently in mono- and dual-species configurations. Adequate control in the relative concentration of AgNPs into the PPy support can be considered a promising strategy to circumvent the overdosage of AgNPs and the ecotoxicity of released ions. The combination of components is favored by the low cytotoxicity of the composites, as demonstrated by cell viability above 70%; the assay revealed potential for the development of various formulations. Additionally, further investigations into the biocompatibility of the composites in different cell lines and their ecotoxicity are necessary to ensure the safety and sustainability of these materials in biomedical applications.

## Materials and Methods

### Maintenance of Bacterial Cultures and Preparation of Inoculum

*S. aureus* (ATCC 25923) and *E. coli* (ATCC 25922) strains were streak plated from −80 °C glycerol stocks onto Tryptic soy agar (TSA) (Merck) and grown at 37 °C for 24 h. Subsequently, to prepare the inoculum, colonies were subcultured in artificial urine medium (AUM),^71^ and it was incubated at 37 °C, 150 rpm, for 16–18 h. Afterward, the cell concentration was assessed by measuring the optical density (OD) at 620 nm, which estimates viable cell density in the suspension. The inoculum was diluted in AUM medium to reach a final 10^6^ CFU/mL concentration.

### Synthesis of PPy, AgNPs, and PPy/AgNPs

Polypyrrole (PPy) was synthesized through chemical polymerization, as reported by da Silva et al.^27^ Silver nanoparticles (AgNPs) were obtained as a solution by chemical synthesis using sodium citrate (Na_3_C_6_H_5_O_7_) (Dinâmica, Brazil) as a reducing agent.^72^ The PPy/AgNPs composite synthesis was based on the previously mentioned procedure for PPy. With this aim, 100 mL of the AgNPs solution was used instead of ultrapure water, followed by polypyrrole polymerization in the media. After the synthesis procedure, the solutions were frozen for 24 h and then lyophilized under vacuum for 24 h at −31 °C and 133.3 Pa in a lyophilizer Enterprise (Terroni, Brazil). Composite stock solutions were prepared, respectively, at the following concentrations: 16.1 μg/mL (PPy), 1.93 μg/mL (AgNPs), and 10.71 μg/mL (PPy/AgNPs) with composites diluted in sterile ultrapure water intercalated by two-step processes of ultrasonic bath for 5 min, interspersed with 1 min of vortexing.

### Characterization Techniques

Fourier transform infrared spectroscopy (FTIR) experiments were conducted using the Fourier IR Prestige-21 spectrometer (Shimadzu). The samples were prepared using a potassium bromide (KBr).^73^ The kinetics of silver nanoparticle formation was evaluated by measuring the plasmonic band on a Hach DR5000 UV–vis spectrometer in the 200–800 nm range, with a 1 nm interval. This technique exploits the unique optical properties of silver nanoparticles, which exhibit a characteristic absorbance peak in the UV–vis spectrum due to the collective oscillation of surface electrons (plasmons) when exposed to light. Tracking changes in absorbance at specific wavelengths, typically around 400–450 nm.^74^ Particle size distribution was determined using a Malvern Zetasizer Nano-ZS90 particle analyzer employing dynamic light scattering (DLS) to measure the size and uniformity of particles suspended in a liquid medium.^75^

### Determination of Biofilm Prevention Concentration (BPC)

The biofilm prevention concentration (BPC) is a relevant parameter for reducing cellular density and preventing biofilm formation.^76^ Thus, based on the design of this study, BPC was used to determine the initial working concentrations. With this aim, different concentrations of PPy and PPy/Ag (100 to 1000 μg/mL) and AgNPs (20 to 90 μg/mL) were evaluated. Briefly, 10 μL of the test concentrations were added into wells of the same column on 96-well sterile flat-bottom plates (Orange Scientific, Braine-l’Alleud, Belgium), followed by incorporation of 190 μL of bacterial inoculum at 10^6^ CFU/mL, prepared with AUM medium. The negative control contained AUM medium, and the positive control contained bacterial inoculum. After 24 h of incubation at 37 °C, three random wells from each column were washed and filled with saline solution (200 μL) and scraped with a micropipette tip to detach the adhered biofilm. The contents of the wells were diluted (1:10), and 10 μL aliquots were plated on TSA medium and incubated for 24 h at 37 °C. BPC_90_ was defined as the lowest concentration of the composite that reduced the number of CFU by at least 90% compared to the positive control.^47,48^ The analyses were conducted in three independent experiments and triplicates for each condition. The highest BPC of each composite was defined as the reference point for biofilm studies, and concentrations of 1/2× BPC and 1× BPC were applied in the following experiments.

### Antibiofilm Activity on Silicone Surface

The study of the antibiofilm effect of PPy, AgNPs, and PPy/AgNPs on silicone surfaces for single and dual-species biofilms involving *E. coli* and *S. aureus* composites made according to Lemos et al.^77^ For this, silicone coupons 1 × 1 cm (Neves & Neves Ltd.a, Porto, Portugal) were prepared as described by Azevedo et al.^50^ The coupons were glued to the bottom of the wells of 24-well tissue culture plates (Orange Scientific) and sterilized in a laminar flow hood under UV light for 30 min. The biofilm cultivation procedure was performed briefly: aliquots of each composite were added, followed by the bacterial inoculum at 10^6^ CFU/mL AUM up to a volume of 1.5 mL. For dual-species biofilms (*E. coli*/ *S. aureus*), inocula were applied in equal volumes (1:1) for each bacterial culture. Controls were defined as positive, containing only bacterial cells, and negative, containing only AUM. The plates were incubated in static conditions at 37 °C for 24 h. Three independent experiments in triplicate were performed for each condition.^22,50^

### Biofilm Removal and Analysis

#### Colonies Forming Unity Determination

After the incubation period ended, the culture medium was carefully removed to avoid breaking the coupon biofilm. The wells were washed once with 1.5 mL of sterile saline solution (0.85% v/v), and the coupons were transferred to Falcon tubes with 5 mL of sterile saline. The tubes were subjected to three-step vortexing for 30 s, followed by sonication for 30 s to remove adhered cells (conditions previously optimized to avoid cell lysis).^22^ Then, 100 μL of the solutions from the tubes were diluted in a series of 1:10 and plated in triplicate in the respective culture media. TSA (Merck) was used for single-species biofilms. For dual-species biofilm, Mannitol Salt Agar (MSA), a selective medium for isolating *Staphylococcus*, and MacConkey Agar (MAC), a differentiating medium for Gram-negatives strains, both manufactured by Liofilchem, Roseto degli Abruzzi, Italy, were utilized. All culture media were prepared according to the manufacturer’s recommendations. The plates were incubated at 37 °C for 24 h. CFUs were quantified in the range between >10 and <100, and the values obtained were converted into log CFU/cm^2^.^50^

### Total Cells by Flow Cytometry

According to standardized protocols, total cell numbers were determined using flow cytometry (cytoFLEX V0-B3-R1, Beckman Coulter, Brea, CA, USA).^52^ For each biofilm sample, 10 μL was acquired at a 10 μL/min flow rate. Bacterial cells were detected using side scatter (SSC) and forward scatter (FSC) signals. The CytExpert software (version 2.4.0.28, Beckman Coulter, Brea, CA, USA) was employed for graphical analysis and quantification. Results were expressed as total cells per cm^2^, providing a standardized metric for comparing cell densities across different conditions.

### Biofilm Morphology by Scanning Electron Microscopy (SEM)

For SEM observation, dual-species biofilms were grown on silicone coupons following a structured protocol. Initially, samples were washed with phosphate-buffered saline (PBS) at pH 7.2, prepared using KH_2_PO_4_ (Exodus Science, Brazil) at a concentration of 10 mM, NaCl (Dinamica, Brazil) at 137 mM, and KCl (LabSynth Ltd.a., Brazil) at 2.7 mM. After washing, biofilms were fixed with 3% glutaraldehyde (Sigma-Aldrich, Portugal) in PBS for 1 h to preserve cellular structure. Subsequently, samples underwent a dehydration process using an ethanol gradient of 10%, 30%, 70%, and 100% ethanol solutions, with each step lasting 15 min. This gradient ensures complete dehydration to avoid structural distortion during SEM observation. After fixation and dehydration, the coupons were dried in a laminar flow chamber at room temperature (22 ± 2 °C) for 24 h to ensure complete removal of solvents.^53,78^ After drying, the samples were coated with a gold film (120 nm thick) using a metallizer (Quorum Q 150R ES, England) for 12 min, a critical step to increase electronic conductivity during imaging. The morphology and interactions of the biofilms were then observed using SEM/EDX (Tescan VEGA3, Czech Republic) under vacuum conditions with an accelerating voltage of 10 kV.

### Assessment of Cytotoxicity

The in vitro cytotoxicity of PPy, AgNPs, and PPy/AgNPs was evaluated as described by Mosmann,^79^ with some adaptations. Murine fibroblasts (ATCC L929), derived from connective tissue, were initially cultured in Dulbecco’s Modified Eagle’s Medium (DMEM) supplemented with 10% fetal bovine serum (FBS) and antibiotics (penicillin at 100 U/mL and streptomycin at 0.1 mg/mL). The cells were incubated at 37 °C with 5% CO_2_. After two passages, the cytotoxicity assay was initiated by seeding 2 × 10^4^ cells per well in a 96-well plate (Orange Scientific), allowing 24 h of incubation under the same conditions. Stock solutions of PPy, AgNPs, and PPy/AgNPs were prepared at 10,000 μg/mL and filtered through a sterile syringe filter (0.22 μm) to ensure sterility. Subsequently, serial dilutions were prepared at concentrations of 4000, 2000, 500, 100, and 50 μg/mL in DMEM, chosen to represent a wide range of potential cytotoxic effects.

After establishing a cell monolayer, the wells were washed with PBS before adding 100 μL per well of each concentration of PPy, AgNPs, and PPy/AgNPs. The plates were incubated for 24 h. Following incubation, the wells were washed twice with PBS, and 100 μL of MTT solution (3-[4,5-dimethylthiazol-2-yl]-2,5-diphenyl tetrazolium bromide, Sigma-Aldrich) at 1 mg/mL was added. The plate was incubated at 37 °C, protected from light, until the formation of violet formazan crystals (approximately 3 h). After removing the MTT solution, 50 μL of absolute isopropyl alcohol was added to dissolve the crystals. Optical density (OD) readings were obtained using a microplate spectrophotometer (SoftMax Pro 5) at 570 nm.

To validate the assay, positive and negative controls were included: cells treated with 10% dimethyl sulfoxide (DMSO) served as the positive control (indicating maximum cytotoxicity), while untreated cells in DMEM + 10% FBS served as the negative control (representing optimal cell viability). The blank consisted of the reagents used in each assay step without a cell monolayer. Based on these values, the average percentage of cell viability was calculated using the values from the negative control (100% viability) and the blank control, as shown in eq 1:^60^
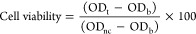
1where OD is the optical density (absorbance) and the subscripts “t,” “b,” and “nc” refer to the test group, blank, and negative control, respectively. Each experiment was performed in triplicate and repeated in three independent assays to ensure reproducibility and accuracy.

### Statistical Analysis

The results were compared using a one-way analysis of variance (ANOVA) and Tukey’s multiple comparisons test. The studies were performed with a 95% confidence level.
